# Suppressing circIDE/miR-19b-3p/RBMS1 axis exhibits promoting-tumour activity through upregulating GPX4 to diminish ferroptosis in hepatocellular carcinoma

**DOI:** 10.1080/15592294.2023.2192438

**Published:** 2023-03-29

**Authors:** Hang Zhai, Sisi Zhong, Runxin Wu, Zhaohong Mo, Shiyang Zheng, Jinhua Xue, Hongyu Meng, Maosheng Liu, Xianyu Chen, Guangquan Zhang, Xiyan Zheng, Fei Du, Ruixi Li, Boxuan Zhou

**Affiliations:** a Department of Quality and Safety Management, The First Affiliated Hospital of Gannan Medical University, Ganzhou, China; b Department of Hepatobiliary Surgery, The Third Affiliated Hospital, Sun Yat-sen University, Guangzhou, China; c Zhongshan School of Medicine, Sun Yat-sen University, Guangzhou, China; d Department of Head and Neck surgery, Cancer Center of Guangzhou Medical University, Guangzhou, China; e Department of Physiology, the School of Basic Medical Sciences of Gannan Medical University, Ganzhou, China; f Department of Gastroentrology, the First Affiliated Hospital of Gannan Medical University, Ganzhou, China; g Department of Hepatobiliary and Pancreatic Surgery, The Eighth Affiliated Hospital, Sun Yat-sen University, Shenzhen, China

**Keywords:** CircRNA, Ferroptosis, RNA binding protein

## Abstract

Ferroptosis is a newly characterized form of iron-dependent non-apoptotic cell death, which is closely associated with cancer progression. However, the functions and mechanisms in regulation of escaping from ferroptosis during hepatocellular carcinoma (HCC) progression remain unknown. In this study, we reported that the RNA binding motif single stranded interacting protein 1 (RBMS1) participated in HCC development，and functioned as a regulator of ferroptosis. Clinically, the downregulation of RBMS1 occurred in HCC tissues, and low RBMS1 expression was associated with worse HCC patients survival. Mechanistically, RBMS1 overexpression inhibited HCC cell growth by attenuating the expression of glutathione peroxidase 4 (GPX4)and further facilitated ferroptosis *in vitro* and *in vivo*. More importantly, a novel circIDE (hsa_circ_0000251) was identified to elevate RBMS1 expression via sponging miR-19b-3p in HCC cells. Collectively, our findings established circIDE/miR-19b-3p/RBMS1 axis as a regulator of ferroptosis, which could be a promising therapeutic target and prognostic factor.

## GRAPHICAL ABSTRACT

**Figure:**
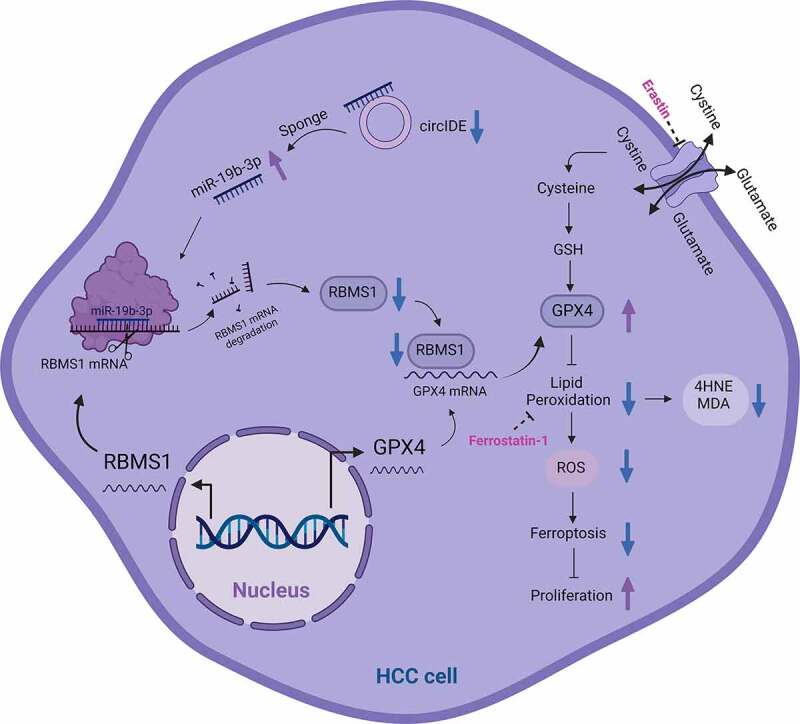

## Introduction

Hepatocellular carcinoma (HCC) is the major primary liver cancer and the leading cause of cancer-related death around the world [1]. Although molecular targeted therapies and immunotherapies have developed rapidly in several decades, there are limited improvements on the prognosis of HCC [2,3]. Ferroptosis is an iron-dependent form of non-apoptotic cell death induced by cysteine depletion and massive lipid peroxidation-controlled membrane damage. Many physiological and pathological diseases are correlative to the dysregulation of ferroptosis [4]. Accumulating evidence has demonstrated that ferroptosis is of significance in HCC progression, and targeting ferroptosis can improve efficacy of sorafenib therapy for HCC [5,6]. Hence, a series of strategies have been developed to promote ferroptosis of cancer cells, including the use of nanomaterials [7] and clinical drugs [8].

There are multiple crucial factors that can regulate ferroptosis [9]. Of them, solute carrier family 7 member 11 (SLC7A11) is responsible for importing extracellular cystine into cells for subsequent conversion to glutathione (GSH), and glutathione peroxidase 4 (GPX4) is the only enzyme capable of suppressing ferroptosis by reducing lipid hydroperoxide using GSH. When SLC7A11-GSH-GPX4 signalling axis is damaged, it causes ferroptosis with the accumulation of the secondary products of lipid peroxidation, such as 4-hydroxynonenal (4HNE) and malondialdehyde (MDA) [10]. However, few studies have focused on the detailed signal transduction and leading regulators of ferroptosis in HCC progression.

RNA-binding proteins (RBPs), interacting with RNAs to form ribonucleoprotein complexes, regulate RNA stability and pre-mRNA splicing [11]. The RNA binding motif single stranded interacting protein 1 (RBMS1) has been reported to play a critical role in tumour progression [12–17]. More importantly, RBMS1 is also identified to modulate the evasion of ferroptosis during lung cancer progression through interacting with the translation initiation factor eIF3d directly to bridge the 3’- and 5’ UTR of SLC7A11. However, whether RBMS1 involved in HCC by regulating ferroptosis-related genes remains to be revealed.

Being a novel transcript, circular RNAs (circRNAs) play an essential role in gene regulation, chromatin modification, genome packaging, and genomic imprinting [18]. Also, circRNAs are proved to get involved in various malignant behaviours of HCC [19,20]. The molecular mechanism of ferroptosis of circRNA, via regulating gene expression as ‘miRNA sponge,’ has been reported in cancers [21,22]. For example, ferroptosis of thyroid cancer cells is inhibited by hsa_circ_0067934 based on miR-19b-3p/SLC7A11 signalling [22]. However, the role of circRNAs in the regulation of ferroptosis during HCC progression needs to be revealed.

In this study, we aimed to explore the expression, functions, and mechanisms of RBMS1 in HCC. We found that RBMS1 was silenced in HCC tissues and RBMS1 downregulation was associated with poor clinical outcomes. Mechanistically, upregulation of RBMS1 inhibited HCC cell growth and promoted ferroptosis by attenuating the expression of GPX4. More importantly, we identified the function of RBMS1 in potentiating ferroptosis and inhibiting tumour growth of HCC, which was regulated by circIDE/miR-19b-3p axis. Collectively, our findings suggested circIDE/miR-19b-3p/RBMS1 axis might be a promising therapeutic target for HCC.

## Materials and methods

### Cell lines and clinical specimens

Human HCC cell lines SMMC-7721, Huh7, Hep3B, and HepG2 and human hepatic cell line LO2 were obtained from Cell Bank of the Chinese Academy of Sciences (Shanghai, China). These cells were maintained in a medium as previously described [23]. All human HCC tissue specimens were obtained from the First Affiliated Hospital of Gannan Medical University from 2014 to 2016. The study patients signed informed consents, and the research project was approved by the Ethics Committee of the First Affiliated Hospital of Gannan Medical University (LLSC-2022052501).

### RNA extraction and real-time quantitative polymerase chain reaction (RT-qPCR)

RNA extraction was performed as previously described [24]. For RT-qPCR, mRNA and circRNA were reverse transcribed through QuantiTect Reverse Transcription Kit (Cat#205310, QIAGEN). miRNA was reverse transcribed using Fast miRNA Reverse Transcription Kit (Cat#miR002, ESscience). The method of 2^−ΔΔCt^ was used to calculate the relative RNA expression levels, and a nonparametric method was used to analyse the statistical significance between the two sets of 2^^−ΔΔCt^ values. The primer sequences are listed in **Supplementary Table S1.**

### Western blot analysis

Protein samples were extracted from tissues and cells using RIPA buffer, resolved by SDS-PAGE, and transferred to polyvinylidene fluoride membranes. Afterwards, membranes were blocked with 5% milk and then incubated with the corresponding primary antibodies against RBMS1 (1:1000, Cat#11061–2-AP, Proteintech), GPX4 (1:1000, Cat#A1933, ABclonal), 4HNE (1:1000, Cat#MBS808701), SLC7A11 (1:1000, Cat#A2413, Abclonal), and GAPDH (1:10000, Cat#A19056, ABclonal) at 4°C overnight. Horseradish peroxidase (HRP)-conjugated secondary antibody was incubated for 1 hour at room temperature, and then antigen–antibody reaction signal was imaged using Odyssey_®_ infrared Imaging System (LI-COR Biosciences).

### Immunohistochemistry staining

Paraffin-embedded samples were sectioned at 4 µm thickness, afterwards deparaffinize paraffin sections in xylene, and then hydrate slides in 100%, 95%, 85%, 75%, 50% ethanol, 5 minutes each. Antigen retrieval was performed in 0.01 M citrate buffer or Tris-EDTA buffer (pH = 9) using a pressure cooker for 3 minutes. Blocking the sections in PBS with 5% BSA for 25 min at room temperature and then incubating the sections with antibodies specific for RBMS1 (1:100, Cat#11061–2-AP, Proteintech), GPX4 (1:200, Cat#A1933, ABclonal), 4HNE (1:100, Cat#MBS808701), and Ki67 (1:100, Cat#16667, Abcam) overnight. Finally, the immunodetection was conducted via DAB according to the manufacturer’s protocol.

For calculating the immunohistochemistry staining, 10 random fields with 40×objective were used. RBMS1, GPX4, or 4HNE following the criteria: 0 (no staining), 1 (weak staining), 2 (moderate staining), and 3 (strong staining) was used to calculate staining intensity. For IHC score, it

was determined by multiplying the staining intensity score and percentage of cells that had staining for proteins as previously described [25]. The average score was calculated for each sample. The final IHC score was ranged from 0 (minimum score, 0% cell staining) to 300 (maximum score, 100% cell staining at 3+ intensity).

### Cell counting kit-8 (CCK8) assay

The proliferation rate of HCC cells was assessed by the CCK-8 assay as previously described [26]. Approximately 5 × 10^3^ HCC cells were seeded into 96-well cell culture plates with three replicate wells. Then, 10 µl of the CCK-8 reagent was added into each well. Finally, the absorbance at 450 nm was measured using a multifunctional microplate reader after incubation for 2 hours at 37°C.

For cell viability assay, HCC cells were seeded in 96-well plates and treated with drugs for an appropriate time on the second day. Then, the medium with drugs was removed and replaced with fresh medium containing 10% CCK8 reagent. After incubation for 2 h at 37°C, the absorbance at 450 nm was measured using multifunction microplate reader.

### Colony formation assay

The capability of single cell to form a larger colony *in vitro* was assessed by a colony formation assay as previously described [27]. Briefly, 300 living HCC cells were seeded per well on six well plates and maintained in a complete culture medium for 8–12 days until formation of visible colonies. Colonies were washed with PBS twice, then fixed with 4% PFA for 20 minutes, and subsequently stained with 0.5% crystal violet in ethanol for 30 minutes. After washing with running water, the number of colonies was counted.

### EdU proliferation assay

The ability of DNA synthesis was detected by EdU (5’-ethynyl-2’-deoxyuridine) assay using BeyoClick^TM^ EdU Cell Proliferation Kit with Alexa Fluro 488 (Cat#0071S, Beyotime). HCC cells were seeded in 96-well plates at the desired density, then cells were added with 10µM EdU for 2 hours at 37°C before fixation and permeabilization, and EdU staining was performed as previously described [28]. The cell nuclei were stained with Hoechst 33342 for 15 minutes. Finally, the images were captured by fluorescence microscopy.

### Dectection of reactive oxygen species (ROS) and lipid peroxidation assay

HCC cells were cultured in a 24-well plate and then ROS probe 2,’7’-dichlorodihydrofluorescein diacetate (H2DCFDA, 1:1000) for 20 minutes at 37°C in the dark. Subsequently, cells were washed with PBS three times and covered with Hoechst 33342. Fluorescent signals were analysed by Zeiss LSM780 confocal microscopy.

The lipid peroxidation product MDA concentration in cell lysates was assessed using a Lipid Peroxidation Assay Kit (Cat#ab118970, Abcam) as previously described [29,30].

### Subcutaneous HCC mouse model

C57BL/6 mice (4- to 6-week-old male) were fed in a pathogen-free vivarium under standard conditions at the animal care facility at Sun Yat-sen University. Hepa 1–6 cells transduced with RBMS1 or GPX4 or circIDE overexpression lentiviral vectors were subcutaneously injected into the right flank of mice in 100µl of sterile PBS. IVIS images were taken.

### Plasmid’s construction, stable cell line generation, and oligonucleotide transfection

For generation of RBMS1, GPX4 and circIDE overexpression vectors, the full-length ORF sequences of RBMS1, GPX4 and cDNA of circIDE were cloned into pcDNA 3.1(+) and pLO-ciR vectors, respectively. To construct sh-RBMS1, si-RBMS1 sequences were cloned into pLKO.1 vector. To generate stable cell lines, lentiviral containing the above-mentioned vectors was generated in HEK293T cells as previously described [31]. Subsequently, Hepa 1–6 cells were transduced with lentiviral vector and then selected with 2.5 µg/ml puromycin for 2 weeks. The full-length ORF sequences of RBMS1 and GPX4, sequence cDNA of circIDE and oligonucleotide sequences of shRNA are listed in **supplementary Table S2 , 3**.

MiR-19b-3p mimics, inhibitors, and corresponding negative control were designed and synthesized by Tsingke (Beijing, China). The above-mentioned oligonucleotides were transfected by Lipofectamine^TM^ RNAiMAX Reagent (Invitrogen, CA, USA) according to manufacturer’s recommendation. The details of the above-mentioned oligonucleotides of miRNA are listed in **supplementary Table S4**.

### Subcellular fractionation, RNase R digestion, and actinomycin D (ActD) treatment assay

Cytoplasmic and nuclear RNA of HepG2 and Hep3B were isolated using cytoplasmic and nuclear RNA purification Kit (Cat#21000, Norgen Biotek) as previously described [32]. For RNase R digestion assay, total RNA of HCC cells was treated with RNase R (1 U RNase R:1µg of total RNA) and 10× reaction buffer at 37°C for 15 minutes, and then enzyme was inactivated at 70°C for 10 minutes. For ActD assay, 5µg/ml ActD was added into the complete medium of HCC cells, and then HCC cells were collected at indicated time points. The relative RNA expression was analysed using RT-qPCR and normalized to the values measured in 0 hour.

### Biotinylated RNA pull-down assay

Biotinylated RNA pull-down was conducted as previously described [33,34]. The sequence of circRNA probe was designed to bind to specific back-spliced junction of circIDE, whereas the scramble probe was regarded as a negative control. Briefly, 10^7^ HCC cells were lysed in a lysis buffer, and then cell lysates were incubated with 3 µg biotinylated probes or scramble probes for 2 hours at room temperature. Subsequently, the biotin-RNA complex was purified by incubating the lysates with streptavidin magnetic beads (Invitrogen, USA) at 4°C overnight. Finally, the beads were washed with lysis buffer 5 times, and RNA complex-capture beads were extracted with TRIzol reagent for further analysis. The probe sequences for RNA pull-down assay are listed in **supplementary Table S5**.

### Fluorescence *in situ* hybridization (FISH)

The subcellular localization of circIDE and miR-19b-3p was identified by FISH as previously described [35]. Briefly, HCC cells were placed and fixed with 4% formaldehyde in 24-well plates containing climbing pieces. After permeabilized in PBST (PBS contains 0.05% Triton X-100) and dehydrated by ethanol, cells were prehybridized in 2× SSC with 50% formamide for 1 hour at 55°C and subsequently hybridized with 20 nM FAM-labelled circIDE probes and Cy5-labelled miR-19b-3p probes at 55°C overnight in hybridization buffer (50% formamide, 2× SSC). DAPI (Life Technology, USA) was used to label nuclei. The fluorescent images were captured by laser scanning confocal microscopy (LSM800, Zeiss). The sequences of circIDE and miR-19b-3p probes are listed in **supplementary Table S6**.

### Luciferase reporter assay

Luciferase reporter assay was performed using the Dual-Luciferase Reporter Assay System (Promega, USA) as described earlier [36]. In brief, HCC cells were transfected with luciferase reporter constructs, miR-19b-3p mimic, miR-19b-3p inhibitor, and Renilla luciferase using Lipofectamine ^TM^ 3000 Reagent (Invitrogen, USA). After transfection, firefly luciferase and Renilla luciferase activity were measured to calculate the luciferase activity.

### Bioinformatic and statistical analysis

For bioinformatic analysis, the possible miRNAs that targeting RBMS1 were predicted using four online databases, including miRmap (https://mirmap.ezlab.org/), PicTar (https://pictar.mdc-berlin.de/), TargetScan (https://www.targetscan.org/vert_80/), and PITA (https://tools4mirs.org/software/target_prediction/pita/). MiRNA-sequencing profiles were downloaded from the TCGA dataset (http://portal.gdc.com), and statistical analysis and ggplot2 (v3.3.2) were completed using R program v4.0.3; *P* value <0.05 was considered statistically significant. The potential circRNAs acted as miR-19b-3p sponge were predicted using Starbase (https://starbase.sysu.edu.cn/). Additionally, GSE155949 and GSE156088 were downloaded from the GEO database, and differential circRNAs expression analysis was conducted by the R project.

Statistical analysis was performed through SPSS 19.0 (IBM, Chicago, USA) and GraphPad (Prism ver.7, La Jolla, CA, USA). Two-tailed Student’s *t*-test was used to compare two groups. One-way analysis of variance (ANOVA) was used for multiple conditions compared to one variable. *P* value <0.05 was considered to be of statistical significance.

## Results

### RBMS1 is downregulated in HCC and low expression of RBMS1 correlates with poor HCC patient survival

**Figure 1.**
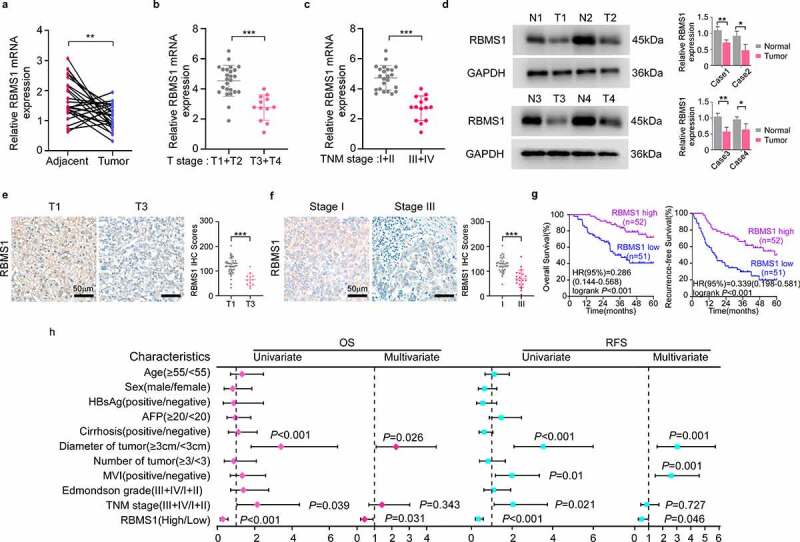
RBMS1 is downregulated in HCC and low expression of RBMS1 correlates with poor HCC patient survival. **(a)** RT-qPCR analysis of RBMS1 expression in 25 cases paired HCC tissues and adjacent liver tissues. **(b)** Comparison of RBMS1 expression between patients with T stage 1–2 (T1+T2, *n*=25) and T stage 3–4 (T3+T4, *n*=12), detected by RT-qPCR. **(c)** Comparison of RBMS1 expression between patients with TNM stage I-II (*n*=23) and TNM stage III-IV (*n*=14), detected by RT-qPCR. **(d)** Western blotting analysis of RBMS1 expression in HCC tissues and normal liver tissues. (**e, f)** Representative images and statistics of IHC staining of RBMS1 in HCC tissue of T stage T1 and T3 and clinical TNM stage I and III. Scale bar, 50 µm. **(g)** Kaplan–Meier analysis of correlation between RBMS1 expression and OS, RFS. **(h)** Forrest plot of univariate or multivariate Cox proportional hazard regression indicated the impact of different characteristics on OS and RFS. Data are denoted as mean ± SD from three independent experiments.

To characterize the function of RBMS1 in HCC, we first searched the expression of RBMS1 in HCC tissues and paired adjacent normal liver tissues by RT-qPCR. We found that RBMS1 expression was higher in adjacent normal liver tissues than in HCC tissues (Figure 1a). In addition, the expression of RBMS1 was declined with tumour staging (T stage) progress and advanced clinical TNM stage (Figure 1b,c). Furthermore, western blotting and immunohistochemistry (IHC) staining results consistently showed that RBMS1 expression was lower in HCC tissues, particularly in higher T stage and advanced clinical TNM stage (Figure 1d-f). To investigate the correlation between RBMS1 expression and the clinicopathological characteristics of HCC patients, we divided 103 HCC patients into high- and low-expression groups on the basis of the median value of RBMS1 IHC staining. We found that the expression level of RBMS1 was closely related to tumour number, tumour size, and TNM stage **(supplementary Table S7)**. Moreover, Kaplan–Meier survival analysis revealed that patients with low RBMS1 expression had worse OS and RFS (Figure 1g). Univariate and multivariate analysis indicated that low expression of RBMS1 was an independent prognostic factor for OS and RFS in HCC (Figure 1h). Collectively, these results indicate that RBMS1 is involved in HCC progression and low RBMS1 expression is associated with an unfavourable prognosis for HCC.

*P < 0.05; **P < 0.01; ***P < 0.001.

### RBMS1 controls the mRNA stability of GPX4 to mediate ferroptosis in HCC

**Figure 2.**
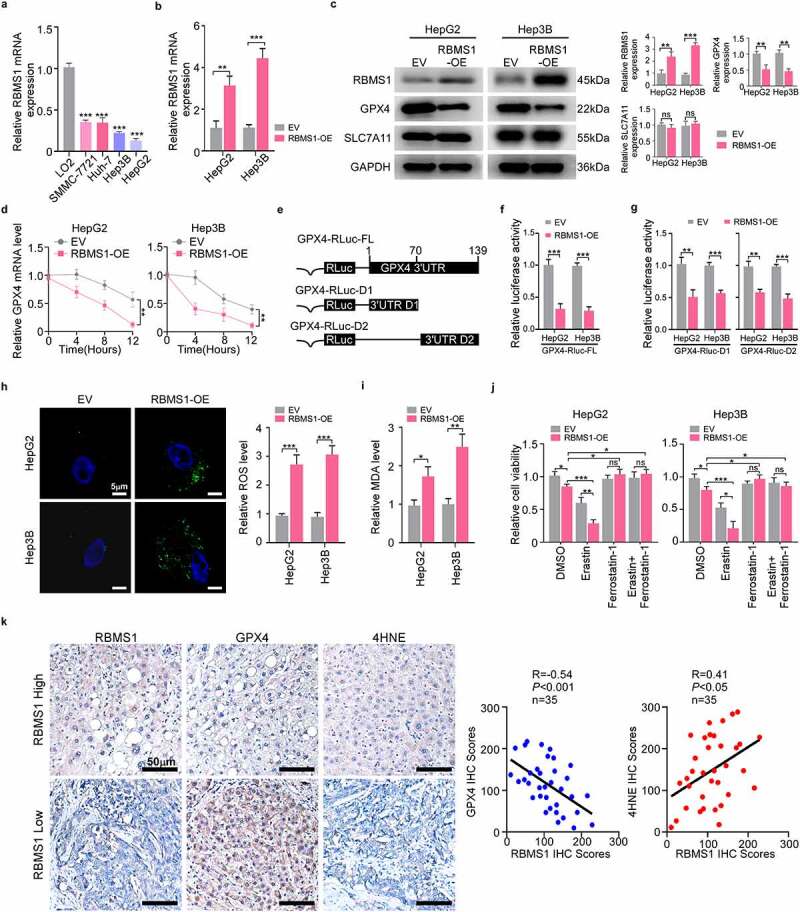
RBMS1 overexpression promotes ferroptosis in HCC cells. **(a)** RT-qPCR analysis for the expression of RBMS1 in normal liver cell line and HCC cell lines. **(b)** RT-qPCR analysis of efficiency of RBMS1 overexpression in HepG2 and Hep3B cell lines, compared to empty vector. **(c)** Western blotting of analysis of RBMS1, GPX4, and SLC7A11 expression in HepG2 and Hep3B as indicated treatments. **(d)** Half-life of GPX4 mRNA after treatment with ActD in HepG2 and Hep3B cells with or without RBMS1 overexpression. **(e)** Schematic of GPX4 luciferase reporter plasmids. GPX4-RLuc-FL (the full length of 3’-UTR); GPX4-RLuc-D1 (1–69 nt region of 3’UTR); and GPX4-RLuc-D2 (70–139 nt region of 3’UTR). **(f-g)** The relative luciferase activity of HepG2 and Hep3B cells with or without RBMS1 overexpression after transfecting GPX4-RLuc-FL **(f)**, GPX4-RLuc-D1, and GPX4-RLuc-D2 **(g)** luciferase reporter vectors. **(h)** Representative images of H2DCFDA staining (green) and quantification of ROS level in HepG2 and Hep3B cells. Scale bar, 5 µm. **(i)** The assessment of MDA level in HepG2 and Hep3B cells. **(j)** Relative cell viability of HepG2 and Hep3B cells with or without RBMS1 overexpression after treated with erastin, ferrostatin-1, or both combined compared to corresponding control group. **(k)** Immunohistochemistry staining and correlation analysis of protein levels of RBMS1, GPX4, and 4HNE in clinical HCC samples. Scale bar, 50 µm. Data are denoted as mean ± SD from three independent experiments.

To detect the RBMS1 level in a panel of HCC cell lines (SMMC-7721, Huh7, Hep3B, and HepG2) and normal hepatic cell line LO2, we found that RBMS1 was strikingly decreased in HCC cell lines by RT-qPCR (Figure 2a). Subsequently, we performed functional experiments by overexpressing RBMS1 in HepG2 and Hep3B cells. RT-qPCR and western blotting were performed to verify the transfection efficiency (Figure 2b,c). Given that RBMS1 affected cell growth by ferroptosis-dependent manner in lung cancer [17], we evaluated the effect of RBMS1 on ferroptosis of HCC cells. We then detected the expression of ferroptosis regulators by western blotting and found that RBMS1 overexpression diminished the GPX4 expression, while there was no effect on SLC7A11 expression in HCC cells (Figure 2c). We further testified whether RBMS1 regulated the mRNA levels of GPX4 by modulating the stability of GPX4 mRNA. Based on the treatment of RBMS1-overexpressed HCC cells with actinomycin D, we found that overexpression of RBMS1 remarkedly facilitated the degradation of GPX4 mRNA (Figure 2d). Moreover, we constructed luciferase reporters that contained the full-length and truncated 3’-UTR of GPX4, respectively (Figure 2e), showing that the activity of luciferase reporter GPX4-Rluc-FL was distinctly suppressed by overexpressed RBMS1 in HCC cells. Meanwhile, the activity of luciferase reporters GPX4-RLuc-D1 and GPX4-RLuc-D2 was both significantly inhibited in RBMS1-overexpressed cells. These results indicated that both of the truncated 3’UTR portions (D1 and D2) contained multiple RBMS1 binding sites (Figure 2f-g). Hence, our findings demonstrate that RBMS1 regulates the stability of GPX4 mRNA through its 3’-UTR.

*P < 0.05; **P < 0.01; ***P < 0.001.

Furthermore, enforced expression of RBMS1 notably induced the accumulation of ROS and MDA (an end-product of lipid peroxides) (Figure 2h-i). More importantly, RBMS1 overexpression inhibited HepG2 and Hep3B cell viability, which was strengthened and prevented by erastin (ferroptosis activator) and ferrostatin-1 (ferroptosis inhibitor), respectively, indicating that RBMS1 hindered HCC cell proliferation by ferroptosis (Figure 2j). To further investigate the correlation between RBMS1 and ferroptosis of HCC, we performed IHC staining to evaluate RBMS1, GPX4, and 4HNE expression in clinical HCC tissues (Figure 2k). We confirmed the negative correlation between RBMS1 and GPX4 expression by quantifying the IHC staining of 35 cases HCC tissues. In agreement, the positive correlation between RBMS1 and 4HNE expression was also observed (Figure 2k). Taken together, our findings demonstrate that RBMS1 overexpression decreases the mRNA stability of GPX4 to promote ferroptosis in HCC cells.

### GPX4 rescues the inhibiting proliferation effect of HCC cells induced by RBMS1 overexpression *in vitro* and *in vivo*

**Figure 3.**
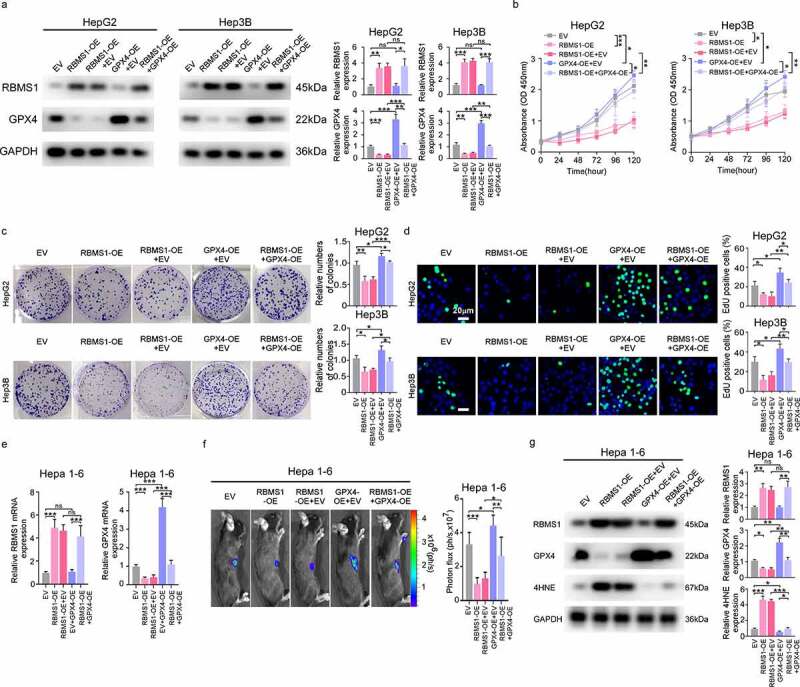
GPX4 rescues the inhibiting proliferation effect of HCC cells induced by RBMS1 overexpression *in vitro* and *in vivo*. **(a)** Western blotting analysis of RBMS1 and GPX4 expression in RBMS1-overexpressed HepG2 and Hep3B cells with or without GPX4 overexpression, compared to vector control. **(b-d)** Effects of RBMS1 overexpression with or without GPX4 overexpression on the proliferation of HepG2 and Hep3B cells were, respectively, assessed by CCK-8 assay **(b)**, colony formation assay **(c),** and EdU staining **(d)**, scale bar, 20 µm. **(e-g)** Hepa 1–6 cells (transduced with empty vector lentivirus, or vector overexpressing RBMS1 withor without overexpressing GPX4) were subcutaneously injected into the right flank of C57BL/6 mice. RT-qPCR analysis for the expression of RBMS1 and GXP4 in Hepa 1–6 cells **(e)**. Tumor growth was monitored by Xenogen IVIS 200 imaging system **(f)**. Western blotting analysis for the expression of RBMS1, GPX4, and 4HNE in Hepa 1–6 cells **(g)**. Data are denoted as mean ± SD from three independent experiments.

To further investigate the combined biological effects of RBMS1 and GPX4, we transfected GPX4 overexpression plasmid into RBMS1 upregulated HepG2 and Hep3B cells. Western blotting showed that RBMS1 overexpression downregulated the level of GPX4, while GPX4 overexpression rescued the decreased expression of GPX4 in RBMS1 overexpressed HepG2 and Hep3B cells (Figure 3a). Additionally, the proliferation of HepG2 and Hep3B cells suppressed by RBMS1 overexpression could be restored after transfection of GPX4 overexpression plasmid through CCK8, colony formation, and EdU staining assays (Figure 3b-d). In line with the results observed *in vitro*, overexpressing GPX4 reversed the decreased GPX4 expression caused by RBMS1 overexpression by RT-qPCR, bioluminescent signals in C57BL/6 mice incubated hepa 1–6 cells were remarkably lower in the RBMS1 overexpression group than in the vector group, while the effects of RBMS1 were further mitigated by GPX4 overexpression (Figure 3e-f). Then, we analysed the expression of RBMS1, GPX4, and 4HNE in mice xenograft tumours. Consistently, we found that RBMS1 overexpression notably decreased GPX4 expression and increased 4HNE level, while the effects of RBMS1 were reversed after overexpression of GPX4 (Figure 3g). Taken together, these results confirm that GPX4 rescues the inhibiting proliferation effect of HCC cells induced by RBMS1 overexpression *in vitro* and *in vivo*.

*P < 0.05; **P < 0.01; ***P < 0.001.

### MiR-19b-3p inhibits ferroptosis and potentiates proliferation of HCC cells by repressing RBMS1 expression

**Figure 4.**
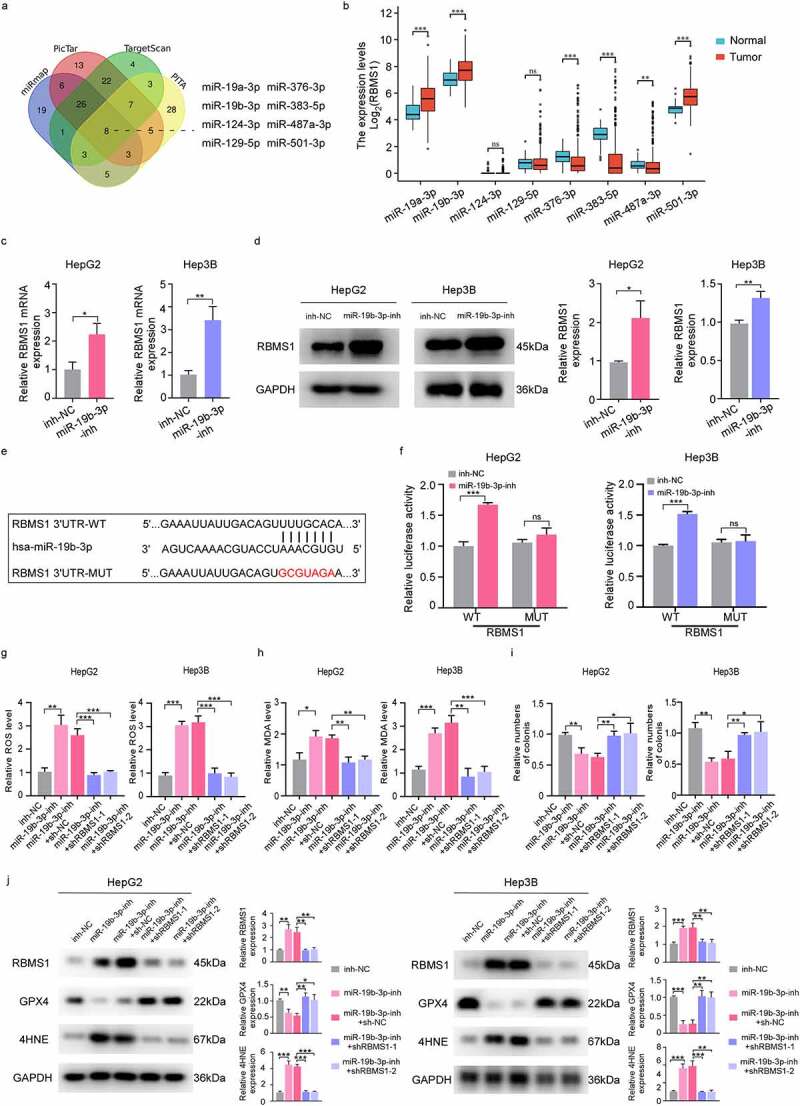
MiR-19b-3p inhibits ferroptosis and potentiates proliferation of HCC cells by repressing RBMS1 expression. **(a)** Prediction of miRNAs targeting RBMS1 by miRmap, PicTar, TargetScan, and PITA databases. **(b)** The expression of miR-19a-3p, miR-19b-3p, miR-124-3p, miR-129-5p, miR-376-3p, miR-383-5p, miR-487a-3p, and miR-501-3p in HCC and normal liver tissues through TCGA database. **(c, d)** RT-qPCR and western blotting analysis for the expression of RBMS1 in HepG2 and Hep3B cells after transfecting miR-19b-3p inhibitor. **(e)** Schematic diagram of luciferase reporter vector containing wild-type (WT) or mutant (MUT) putative miR-19b-3p binding sites of the 3’ UTR of RBMS1. **(f)** The dual luciferase reporter assays demonstrated that miR-19b-3p inhibitor influenced the luciferase activity of luciferase reporter vectors containing WT or MUT 3’UTR of RBMS1. **(g, h)** RBMS1 knockdown plasmids were added to the basis of miR-19b-3p inhibitor transfection in HepG2 and Hep3B cells. The ROS and MDA level were measured as indicated treatments. **(i)** Proliferation of HepG2 and Hep3B cells as indicated treatments was evaluated by colony formation assay. **(j)** RBMS1, GPX4, and 4HNE expression in HepG2 and Hep3B as indicated treatments was evaluated by western blotting. Data are denoted as mean ± SD from three independent experiments.

Increasing reports have revealed that miRNA-mRNA regulatory axis is associated with many cancers [37]. To further explore the potential target miRNAs of RBMS1, we analysed four publicly available datasets including miRmap, PicTar, TargetScan, and PITA, and eight potential miRNAs were identified (Figure 4a). Furthermore, we found that the expression of miR-19a-3p, miR-19b-3p, and miR-501-3p was significantly increased in HCC tissues compared to adjacent normal tissues from TCGA database (Figure 4b). Given that miR-19b-3p facilitated tumour progression in multiple cancers [38,39], therefore, we focused on miR-19b-3p for further analysis. Moreover, the mRNA and protein levels of RBMS1 were increased in HepG2 and Hep3B cells transfected with miR-19b-3p inhibitor (Figure 4c,d). Additionally, wild-type RBMS1 (RBMS1-WT) or mutant RBMS1 (RBMS1-MUT) was cloned into reporter vectors (Figure 4e). Luciferase reporter assays showed that miR-19b-3p inhibitor obviously elevated luciferase activity of RBMS1-WT reporter vector but not in the RBMS1-MUT reporter vector in HCC cells (Figure 4f). We further examined whether miR-19b-3p inhibited HCC cell ferroptosis by downregulating RBMS1. Our results demonstrated that miR-19b-3p inhibitor induced ROS and MDA accumulation, while the effects of miR-19b-3p inhibitor were rescued by knocking down RBMS1 (Figure 4g,h). Furthermore, colony formation assay showed that miR-19b-3p inhibitor significantly suppressed HCC cell proliferation, and the effects of miR-19b-3p inhibitor were also blocked by silencing RBMS1 (Figure 4i). Meanwhile, we found that GPX4 level was decreased after transfection of miR-19b-3p inhibitor in HepG2 and Hep3B cells, while the influences of miR-19b-3p inhibitor on GPX4 expression were blocked by RBMS1 knockdown. However, the expression of RBMS1 and 4HNE was distinctly increased after transfection of miR-19b-3p inhibitor while reversed by additional RBMS1 knockdown treatment in HepG2 and Hep3B cells (Figure 4j). Hence, these results demonstrate that miR-19b-3p inhibits ferroptosis and promotes proliferation of HCC cells through curbing RBMS1 expression.

*P < 0.05; **P < 0.01; ***P < 0.001.

### CircIDE directly binds to miR-19b-3p in HCC cells

**Figure 5.**
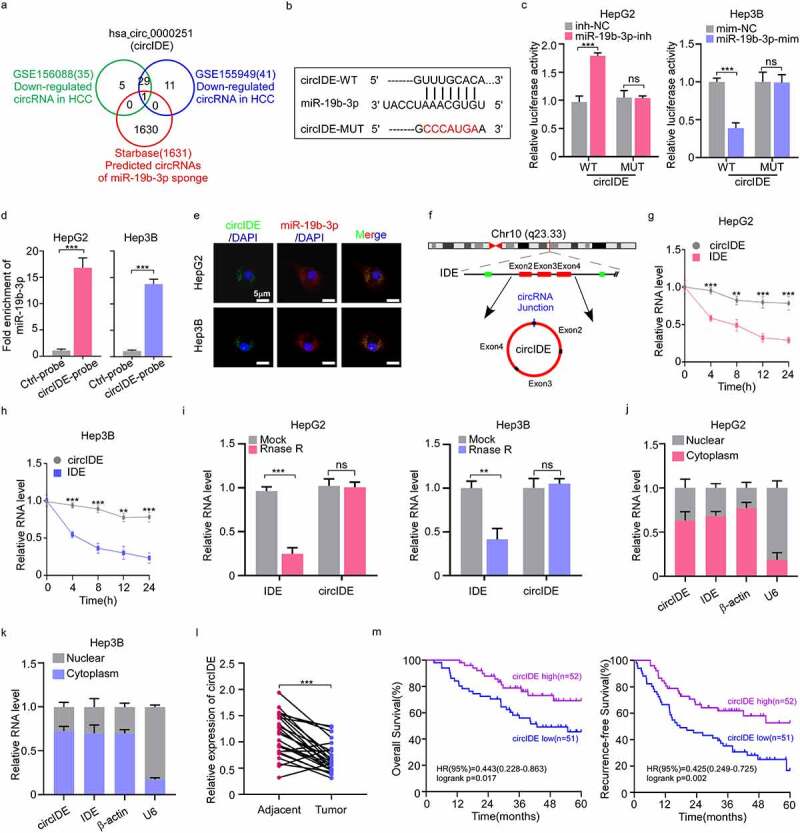
CircIDE directly binds to miR-19b-3p in HCC cells. **(a)** Identification the potential circRnas acted as miR-19b-3p sponge through Starbase, GSE155949 and GSE156088 datasets. **(b)** Schematic diagram of luciferase reporter vectors containing wild-type (WT) or mutant (MUT) putative miR-19b-3p binding sites of circIDE. **(c)** The relative luciferase activity of HepG2 and Hep3B cells co-transfected with WT or MUT circIDE luciferase reporter vectors and miR-19b-3p inhibitor or mimic. **(d)** CircRNA pull-down using biotin-coupled circIDE compared to control-biotin. MiR-19b-3p expression was detected by RT-qPCR. **(e)** Co-localization of circIDE and miR-19b-3p were detected by FISH assay. Scale bar, 5 µm. **(f)** Genomic loci of the IDE gene and circIDE blue dot indicated the back-splicing of IDE exon 2 to exon 4. **(g-h)** Half-life of IDE and circIDE after treatment with ActD in HepG2 and Hep3B cells. **(i)** Relative expression of IDE and circIDE in HepG2 and Hep3B cells with or without RNase R treatment was examined by RT-qPCR. **(j-k)** Results of cytoplasmic and nuclear RNA fraction assays. β-actin and U6 were used as cytoplasmic and nuclear positive controls, respectively. **(l)** RT-qPCR analysis of circIDE expression in HCC tissues and paired adjacent normal liver tissues (*n*=25). **(m)** Kaplan–Meier analysis of correlation between circIDE expression and OS, RFS. Data are denoted as mean ± SD from three independent experiments.

The function and mechanism of circRNAs acted as miRNA sponges in the regulation of HCC progression are still largely unknown. To determine the possibility that circRNAs act as miR-19b-3p sponge, we identified downregulated circRNAs from GSE156088 and GSE155949 of HCC tissues, in contrast to tumour-adjacent tissues, and further screened circRNAs with miR-19b-3p binding sites from Starbase. We identified hsa_circ_0000251 (termed as circIDE) containing miR-19b-3p binding sites (Figure 5a). We also inserted circIDE wild-type (WT) and mutant (MUT) binding site sequences to further construct luciferase reporter vectors (Figure 5b) and found miR-19b-3p inhibitor and mimic remarkably increased and decreased the luciferase activity of luciferase reporter plasmid with circIDE-WT, respectively, while there was no change for circIDE-MUT (Figure 5c). To further validate circIDE interacted with miR-19b-3p, we conducted circRNA pull-down assay and the results showed that miR-19b-3p was enriched in the circIDE-captured faction in comparison to the negative control (Figure 5d). Moreover, fluorescence *in situ* hybridization (FISH) assay showed that circIDE was directly bound to miR-19b-3p (Figure 5e).

*P < 0.05; **P < 0.01; ***P < 0.001.

CircIDE was evolved from exon 2 to exon 4 of a protein-coding gene IDE (Figure 5f). To investigate the stability of circIDE and IDE mRNA, we treated HepG2 cells and Hep3B cells with actinomycin D, which can suppress transcription, showing that linear IDE mRNA was less stable compared to circIDE (Figure 5g,h). Additionally, we found that the resistance of circIDE to RNase R exonuclease digestion further supported the circular structure (Figure 5i). Moreover, circIDE has preference for its localization in the cytoplasm (Figure 5j,k). In accordance with the circRNA datasets (GSE156088 and GSE155949) results, circIDE level was markedly lower in human HCC tissues compared to paired tumour-adjacent tissues (Figure 5l). Kaplan–Meier survival analysis further demonstrated that patients with high expression of circIDE had better OS and RFS (Figure 5m). In summary, these observations indicate that circIDE acted as miR-19b-3p sponge is downregulated in HCC tissues and that the low circIDE expression is corresponded with unfavourable survival.

### CircIDE enhances ferroptosis and attenuates proliferation of HCC cells via miR-19b-3p/RBMS1 axis

**Figure 6.**
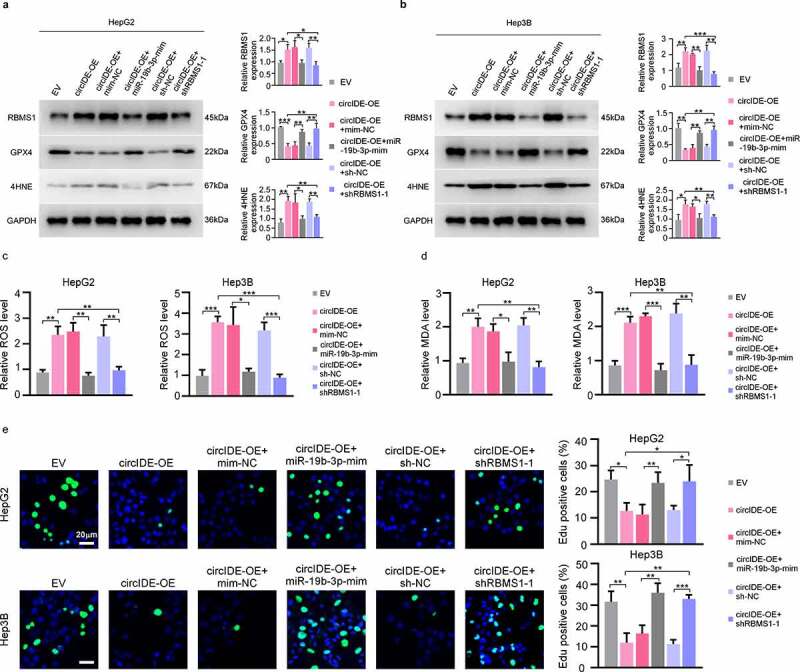
CircIDE enhances ferroptosis and attenuates proliferation of HCC cells via miR-19b-3p/RBMS11 axis. **(a-e)** After circIDE overexpression, HepG2 and Hep3B cells were transfected with miR-19b-3p mimic or RBMS1 knockdown plasmid, respectively. **(a, b)** Western blotting analysis of RBMS1, GPX4, and 4HNE expression in HepG2 and Hep3B as indicated treatments. **(c, d)** the assessment of ROS and MDA level in HepG2 and Hep3B cells as indicated treatments. **(e)** Proliferation of HepG2 and Hep3B cells as indicated treatments was evaluated by EdU staining. Scale bar, 20 µm. Data are denoted as mean ± SD from three independent experiments.

To investigate the impacts of circIDE/miR-19b-3p/RBMS1 axis on ferroptosis and proliferation of HCC cells, we conducted a series of rescue assays. RT-qPCR and western blotting results implicated that the increased RBMS1 level mediated by overexpression circIDE was reversed by the transfection of miR-19b-3p mimic and shRBMS1 plasmid in HepG2 and Hep3B cells, respectively **(supplementary Figure S1a, b，**Figure 6a,b). Additionally, the protein level of 4HNE was consistent with the RBMS1 expression, while the expression of GPX4 was opposite to RBMS1 protein level in the aforementioned rescue experiments (Figure 6a,b). Moreover, our results demonstrated that overexpression of circIDE promoted ROS and MDA accumulation, while the inducing ferroptosis effects of circIDE overexpression were reversed by the transfection of miR-19b-3p mimic and shRBMS1 plasmid in HepG2 and Hep3B cells (Figure 6c,d). Moreover, EdU staining demonstrated that the decreased cell proliferation potentially induced by overexpression circIDE was reversed by the transfection of miR-19b-3p mimic and shRBMS1 plasmid in HepG2 and Hep3B cells, respectively (Figure 6e). Taken together, circIDE enhances ferroptosis and inhibits proliferation of HCC cells via miR-19b-3p/RBMS1 axis.

*P < 0.05; **P < 0.01; ***P < 0.001.

### GPX4 overexpression blocks circIDE induced inhibition of tumour growth *in* *vivo*

**Figure 7.**
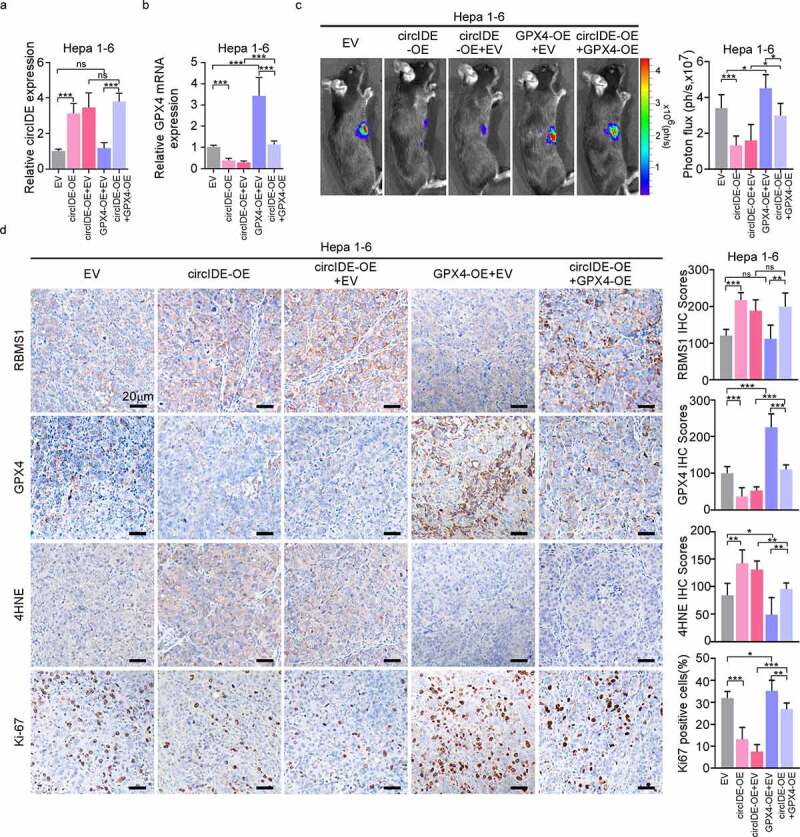
GPX4 overexpression restores circIDE induced inhibition of tumor growth. **(a, b)** RT-qPCR analysis of circIDE and GPX4 expression of circIDE overexpression of Hepa 1–6 cells with or without GPX4 overexpression, compared to vector control. **(c)** Tumor growth was monitored by Xenogen IVIS 200 imaging system. **(d)** Representative IHC staining and corresponding statistics for RBMS1, GPX4, 4HNE and Ki67 expression in the xenografts tumors. Scale bars, 20 µm. Data are denoted as mean ± SD from three independent experiments.

Finally, subcutaneous HCC mouse model was established to assess the combined biological effects of RBMS1 and GPX4 *in vivo*. RT-qPCR validated the transduction efficiency and found that downregulated GPX4 expression by overexpressing circIDE was rescued by additional GPX4 overexpression in hepa 1–6 cells (Figure 7a,b). Moreover, we found that tumour growth was obviously attenuated by overexpression of circIDE, while the effects of circIDE were reversed by additional transduction of GPX4 overexpression lentiviral (Figure 7a-c). Furthermore, IHC staining of mice xenograft tumours demonstrated that circIDE overexpression increased the level of RBMS1 and 4HNE, while Ki67 and GPX4 expression was decreased by circIDE overexpression (Figure 7d). However, the effects of circIDE were further reversed by additional GPX4 overexpression for the expression of RBMS1, Ki67, GPX4, and 4HNE in xenograft tumours. In summary, our results indicate that GPX4 can reverse the circIDE induced inhibition of HCC growth *in vivo*.

*P < 0.05; **P < 0.01; ***P < 0.001.

## Discussion

Regulated by various factors, ferroptosis can be involved in regulating cancer progression [40]. However, the regulator of ferroptosis in HCC has not been widely investigated. In the current study, we uncovered circIDE, as a novel ferroptotic regulatory molecule, mediated RBMS1 expression by sponging miR-19b-3p, thereby inhibiting HCC progression. Additionally, we found that RBMS1 inhibited the expression of GPX4 and subsequently induced ferroptosis and suppressed proliferation of HCC cells. Collectively, our findings demonstrated that circIDE/miR-19b-3p/RBMS1 axis suppressed HCC progression via promoting ferroptosis.

A major characteristic of cancer is capable of escaping from regulated forms of cell death. Unlike apoptosis or necroptosis, ferroptosis, a novel non-apoptotic regulated cell death, is independently free from caspase activity and receptor-interacting protein kinase 1 (RIPK1) activity. The underlying mechanism of ferroptosis, involving the action of divalent iron or lipoxygenase, catalyzes the metabolism of unsaturated fatty acids on cell membranes, leading to lipid peroxidation and ultimately inducing cell death [41].

Accumulating evidence showed that epigenetic regulation exerted an influence on the propensity of cells undergoing ferroptosis. The RNA binding protein (RBP) RBMS1 containing 2 RNA recognition motifs has been shown to bind directly to the C-terminal portion of c-Myc, thereby encouraging the co-transformational activity of c-Myc with Ras [42]. Identified as c-Myc gene single-stranded binding protein, RBMS1 played the role of inhibitor in transcripting alpha-smooth muscle actin (αSMA) gene in chicken visceral smooth muscle cells [43]. Recent studies have shown that increased RBMS1 in lung cancer is proportional to survival in patients. Knocking down RBMS1 suppressed the translation of SLC7A11 and reduced SLC7A11C-mediated cystine uptake and eventually facilitated ferroptosis and sensitized radioresistant lung cancer cells to radiotherapy [17]. However, we found that RBMS1 expression was dramatically decreased in HCC tissues in contrast to adjacent normal samples and forced RBMS1 expression curbed HCC cancer cell proliferation *in vitro and in vivo*. More importantly, RBMS1 overexpression hindered the expression of GPX4 via inhibiting the stability of GPX4 mRNA and then triggered ferroptosis. The distinct expression and functions of RBMS1 might be dependent on the tissue specificity of RBMS1.

Being a crucial regulator of ferroptosis, GPX4 plays a role of phospholipid hydroperoxides and lowers phospholipid hydroperoxide production (AA/AdA-PE-OOH) to the phospholipid alcohol (PLOH), thereby interrupting the lipid peroxidation chain reaction. Moreover, the GPX4 expression is proportional to selenium and GSH [44,45]. At the transcriptional level, the upregulation of GPX4 expression induced by transcription factor AP-2 gamma (TRAP2C) and specificity protein 1 (SP1) with the help of selenium obstructed ferroptosis-associated cerebral haemorrhage [46]. Additionally, erastin-induced GPX4 degradation was promoted by heat shock protein 90 (HSP90)-dependent chaperone-mediated autophagy via identifying its KFERQ-like motif in neuronal cells. However, the mechanism of GPX4 degradation during ferroptosis still remains to be discovered. In our study, we report that the overexpression of RBMS1 represses the expression of GPX4. Although RBMS1 is usually regarded as a post-transcriptional regulator to increase mRNA stability [15], some RNA destabilizing factors may recruit by RBMS1, leading to GPX4 mRNA decay and translational shutdown. Hence, it remains largely elusive whether RBMS1 could associate with RNA destabilizing factor in GPX4 mRNA decay to control ferroptosis.

CircRNAs are closed single-stranded RNAs in which 5’ and 3’ ends are covalently connected by reverse splicing of exons from pre-mRNA. Functionally, circRNAs have been shown to be involved in HCC progression [19,20]. For example, hsa_circ_0001394 plays the role of sponge in promoting HCC progression by modulating miR-527/UBE2A pathway [47]. However, the functions of circRNA in regulating ferroptosis and its underlying mechanisms are still unknown. Our findings reveal that circIDE is downregulated in HCC tissues and low expression of circIDE tends to exhibit poor survival. Mechanically, miR-19b-3p negatively interacts with circIDE, and RBMS1 is shown to be a target of miR-19b-3p. Collective studies demonstrate that circIDE may play the role of sponge in inhibiting HCC progression by being the regulator of the miR-19b-3p/RBMS1 pathway, which affords the great possibility of therapeutic target in HCC treatment.

## Conclusions

Collectively, our findings demonstrate that RBMS1 is downregulated in HCC tissues and low expression of RBMS1 tends to exhibit poor survival. Functional experiments reveal that RBMS1 inhibits proliferation and enhances ferroptosis of HCC cells. We further show that circIDE and miR-19b-3p are the upstream mediators of RBMS1. More importantly, our findings establish circIDE/miR-19b-3p/RBMS1 axis as a regulator of ferroptosis, which could be a promising therapeutic target and prognostic factor.

## Supplementary Material

Supplemental Material

## Data Availability

The datasets presented in this study can be found in online repositories. The names of the repository/repositories and accession numbers can be found at NCBI, GSE155949, and GSE156088. The data in the present study are available from the corresponding author.

## Highlights

- RNA binding protein RBMS1 is downregulated in HCC tissues, and low expression of RBMS1 is related with poor survival;
- Overexpression of RBMS1 inhibits HCC cell proliferation and promotes ferroptosis by downregulating GPX4 *in vitro and in vivo*;
- MiR-19b-3p targets RBMS1 to potentiate HCC cell proliferation and curbs ferroptosis;
- CircIDE acts as a miR-19b-3p sponge to suppress HCC cell proliferation and promote ferroptosis;
- CircIDE/miR-19b-3p/RBMS1 axis acts as a regulator of ferroptosis through affecting the level of GPX4 to modulate HCC progression.
