# Supplementary material for: Suppressing circIDE/miR-19b-3p/RBMS1 axis exhibits promoting-tumour activity through upregulating GPX4 to diminish ferroptosis in hepatocellular carcinoma
Source: Epigenetics. 2023 Mar 29;18(1):2192438. doi: 10.1080/15592294.2023.2192438 (PMC10064926; doi:10.1080/15592294.2023.2192438)
Supplement: Supplemental Material [file KEPI_A_2192438_SM0479.zip › Supplementary files/Supplementary Table and Figure legend.docx]

**Table S1.** The primers (5’-3’) used in this study.

| Primers for RT-qPCR | | | | 5’-3’ |
| --- | --- | --- | --- | --- |
|  | RBMS1 | Forward | AGGTGCAAAGTCCTTCGTGG | |
|  |  | Reverse | GTGACATGGTGTGCTCCATTG | |
|  | miR-19b-3p | Forward | GTGCAAATCCATGCAAAACTGA | |
|  |  | Reverse | GTGCAGGGTCCGAGGTGCT | |
|  | circIDE | Forward | ACAGGGAATCCTAAACACCCC | |
|  |  | Reverse | TCGCTTGTCTTCAGGAGACTT | |
|  | IDE | Forward | GTCCTGTTGTTGGAGAGTTCCCATGTCA | |
|  |  | Reverse | GGGGAATCTTCAGAGTTTTGCAGCCAT | |
|  | β-actin | Forward | GGGAAATCGTGCGTGACATTAAG | |
|  |  | Reverse | TGTGTTGGCGTACAGGTCTTTG | |

**Table S2.** Effective sequences (5’-3’) of lentivirus plasmids-overexpression.

| Plasmids | | 5’-3’ |
| --- | --- | --- |
| LV- RBMS1  LV-GPX4 | ATGGGCAAAGTGTGGAAACAGCAGATGTACCCTCAGTACGCCACCTACTATTACCCCCAGTATCTGCAAGCCAAGCAGTCTCTGGTCCCAGCCCACCCCATGGCCCCTCCCAGTCCCAGCACCACCAGCAGTAATAACAACAGTAGCAGCAGTAGCAACTCAGGATGGGATCAGCTCAGCAAAACGAACCTCTATATCCGAGGACTGCCTCCCCACACCACCGACCAGGACCTGGTGAAGCTCTGTCAACCATATGGGAAAATAGTCTCCACAAAGGCAA  TTTTGGATAAGACAACAAACAAATGCAAAGGTTATGGTTTTGTCGACTTTGACAGCCCTGCAGCAGCTCAAAAAGCTGTGTCTGCCCTGAAGGCCAGTGGGGTTCAAGCTCAAATGGCAAAGCAACAGGAACAAGATCCTACCAACCTCTACATTTCTAATTTGCCACTCTCCATGGATGAGCAAGAACTAGAAAATATGCTCAAACCATTTGGACAAGTTATTTCTACAAGGATACTACGTGATTCCAGTGGTACAAGTCGTGGTGTTGGCTTTGCTAGGATGGAATCAACAGAAAAATGTGAAGCTGTTATTGGTCATTTTAATGGAAAATTTATTAAGACACCACCAGGAGTTTCTGCCCCCACAGAACCTTTATTGTGTAAGTTTGCTGATGGAGGACAGAAAAAGAGACAGAACCCAAACAAATACATCCCTAATGGAAGACCATGGCATAGAGAAGGAGAGGCTGGAATGACACTTACTTACGACCCAACTACAGCTGCTATACAGAACGGATTTTATCCTTCACCATACAGTATTGCTACAAACCGAATGATCACTCAAACTTCTATTACACCCTATATTGCATCTCCTGTATCTGCCTACCAGGTGCAAAGTCCTTCGTGGATGCAACCTCAACCATATATTCTACAGCACCCTGGTGCCGTGTTAACTCCCTCAATGGAGCACACCATGTCACTACAGCCCGCATCAATGATCAGCCCTCTGGCCCAGCAGATGAGTCATCTGTCACTAGGCAGCACCGGAACATACATGCCTGCAACGTCAGCTATGCAAGGAGCCTACTTGCCACAGTATGCACATATGCAGACGACAGCGGTTCCTGTTGAGGAGGCAAGTGGTCAACAGCAGGTGGCTGTCGAGACGTCTAATGACCATTCTCCATATACCTTTCAACCTAATAAG  TTTTGTAATACGACTCACTATAGGGCGGCCGGGAATTCGTCGACTGGATCCGGTACCGAGGAGATCTGCCGCCGCGATCGCCATGAGCCTCGGCCGCCTTTGCCGCCTACTGAAGCCGGCGCTGCTCTGTGGGGCTCTGGCCGCGCCTGGCCTGGCCGGGACCATGTGCGCGTCCCGGGACGACTGGCGCTGTGCGCGCTCCATGCACGAGTTTTCCGCCAAGGACATCGACGGGCACATGGTTAACCTGGACAAGTACCGGGGCTTCGTGTGCATCGTCACCAACGTGGCCTCCCAGTGAGGCAAGACCGAAGTAAACTACACTCAGCTCGTCGACCTGCACGCCCGATACGCTGAGTGTGGTTTGCGGATCCTGGCCTTCCCGTGTAACCAGTTCGGGAAGCAGGAGCCAGGGAGTAACGAAGAGATCAAAGAGTTCGCCGCGGGCTACAACGTCAAATTCGATATGTTCAGCAAGATCTGCGTGAACGGGGACGACGCCCACCCGCTGTGGAAGTGGATGAAGATCCAACCCAAGGGCAAGGGCATCCTGGGAAATGCCATCAAGTGGAACTTCACCAAGTTCCTCATCGACAAGAACGGCTGCGTGGTGAAGCGCTACGGACCCATGGAGGAGCCCCTGGTGATAGAGAAGGACCTGCCCCACTATTTCACGCGTACGCGGCCGCTCGAGCAGAAACTCATCTCAGAAGAGGATCTGGCAGCAAATGATATCCTGGATTACAAGGATGACGACGATAAGGTTTAA | |
| LV-circIDE | TTTCCAAAAAAAGACTTACAGCAAAATGAATAATCCAGCCATCAAGAGAATAGGAAATCACATTACCAAGTCTCCTGAAGACAAGCGAGAATATCGAGGGCTAGAGCTGGCCAATGGTATCAAAGTACTTCTTATCAGTGATCCCACCACGGATAAGTCATCAGCAGCACTTGATGTGCACATAGGTTCATTGTCGGATCCTCCAAATATTGCTGGCTTAAGTCATTTTTGTGAACATATGCTTTTTTTGGGAACAAAGAAATACCCTAAAGAAAATGAATACAGCCAGTTTCTCAGTGAGCATGCAGGAAGTTCAAATGCCTTTACTAGTGGAGAGCATACCAATTACTATTTTGATGTTTCTCATGAACACCTAGAAGGTGCCCTAGACAGGTTTGCACAGTTTTTTCTGTGCCCCTTGTTCGATGAAAGTTGCAAAGACAGAGAGGTGAATGCAGTTGATTCAGAACATGAGAAGAATGTGATGAATGATGCCTGGAGACTCTTTCAATTGGAAAAAGCTACAGGGAATCCTAAACACCCCTTCAGTAAATTTGGGACAG | |

**Table S3.** Effective sequences (5’-3’) of lentivirus plasmids-knockdown.

| Plasmids | 5’-3’ |
| --- | --- |
| sh-NC | CAGAATACTCCTCGTGAGA |
| shRBMS1-1 | CCATATACCTTTCAACCTAAT |
| shRBMS1-2 | CCACAGAACCTTTATTGTGTA |
| shcircIDE-1 | AGGATGGTCCAGGCAACTCCT |
| shcircIDE-2 | GGATGGTCCAGGCAACTCCTA |

**Table S4.** miRNA mimics and inhibitor sequences (5’-3’).

| Name | | 5’-3’ | |
| --- | --- | --- | --- |
| miR-inhibitor-NC | Sense | | AGUGUCUCAUGUUGAUCAUCAUG |
|  | Antisense | | None |
| miR-19b-3p -inhibitor | Sense | | UCAGUUUUGCAUGGAUUUGCACA |
|  | Antisense | | None |
| miR-mimic-NC | Sense | | AUGACAUGAUGACCACAUAACU |
|  | Antisense | | AGUUAUGUGGUCAUCAUGUCAU |
| miR-19b-3p mimic | Sense | | UGUGCAAAUCCAUGCAAAACUGA |
|  | Antisense | | UCAGUUUUGCAUGGAUUUGCACA |

**Table S5.** The probe sequences (5’-3’) for circRNA pull-down assay.

| Probes for circRNA pull-down | 5’-3’ | |
| --- | --- | --- |
| control-biotin | | ATTGTCCGATCGTCTCACGT |
| circIDE-bition | | CATTTAAACCCTGTCAAAGG |

**Table S6.** The probe sequences (5’-3’) for FISH assay

| Probes for FISH assay | 5’-3’ | |
| --- | --- | --- |
| CircIDE | | CAUUUAAACCCUGUCAAAGG |
| miR-19b-3p | | UCAGUUUUGCAUGGAUUUGCACA |

**Table S7.** Clinical characteristics of 103 HCC patients according to RBMS1 expression.

| Variable | Low RBMS1 | | High RBMS1 | | *P* value |
| --- | --- | --- | --- | --- | --- |
|  | No. | % | No. | % |  |
| All cases | 51 |  | 52 |  |  |
| Age, years  ≤ 55 |  |  |  |  | 0.924 |
|  | 25 | 53.7% | 25 | 63.2% |  |
| > 55 | 26 | 46.3% | 27 | 36.8% |  |
| Gender |  |  |  |  | 0.234 |
| female | 9 | 86.4% | 5 | 82.2% |  |
| male | 42 | 13.7% | 47 | 17.8% |  |
| HBsAg positive |  |  |  |  | 0.764 |
| negative | 4 | 80.2% | 6 | 82.1% |  |
| positive | 47 | 19.8% | 46 | 17.9% |  |
| Liver cirrhosis |  |  |  |  | 0.136 |
| without | 24 | 61.1% | 17 | 61.3% |  |
| with | 27 | 38.9% | 35 | 38.7% |  |
| AFP, ng/ml |  |  |  |  | 0.277 |
| ＜20 | 22 | 52.5% | 28 | 54.6% |  |
| ≥20 | 29 | 47.5% | 24 | 45.4% |  |
| No. tumor |  |  |  |  | **0.034*** |
| ＜3 | 38 | 17.3% | 47 | 21.5% |  |
| ≥ 3 | 13 | 82.7% | 5 | 78.5% |  |
| Edmondson’s grade |  |  |  |  | 0.344 |
| I + II | 35 | 71.6% | 40 | 88.3% |  |
| III + IV | 16 | 28.4% | 12 | 11.7% |  |
| Tumor size, cm |  |  |  |  | **0.001*** |
| ＜ 3 | 26 | 17.9% | 42 | 38% |  |
| ≥ 3 | 25 | 82.1% | 10 | 62% |  |
| Microvascular invasion |  |  |  |  | 0.623 |
| absent | 34 | 25.3% | 37 | 54.6% |  |
| present | 17 | 74.7% | 15 | 45.4% |  |
| TNM stage |  |  |  |  | **0.008*** |
| I+II | 37 | 29% | 48 | 62% |  |
| III+ IV | 14 | 71% | 4 | 38% |  |

NOTE: *X*^2^ test was used to test the association between two categorical variables. Abbreviations: AFP, alpha-fetoprotein; HCC, hepatocellular carcinoma; HBsAg, hepatitis B surface antigen. *Statistically significant.

**Supplementary Figure 1**


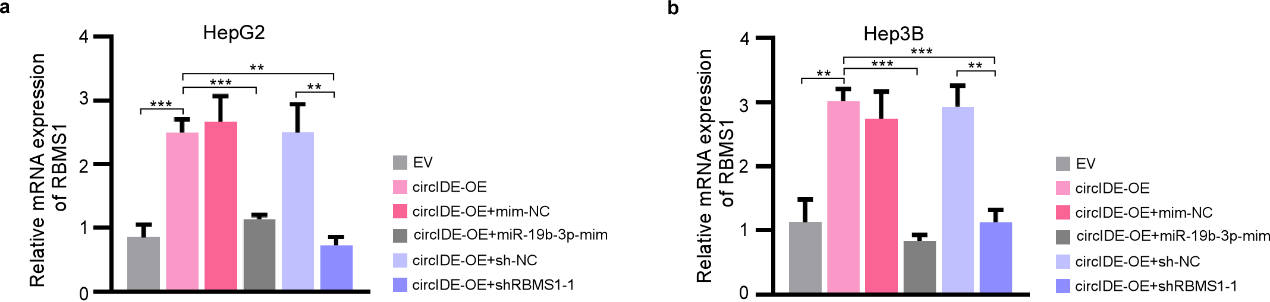


**Supplementary Figure 1**. **CircIDE enhances ferroptosis and attenuates proliferation of HCC cells via miR-19b-3p/RBMS1 axis.** (**a, b)** RT-qPCR analysis of RBMS1 expression in HepG2 and Hep3B cells after transfected with miR-19b-3p mimic or RBMS1 knockdown plasmid respectively after circIDE overexpression.
