# Supplementary figures and images for: Suppressing circIDE/miR-19b-3p/RBMS1 axis exhibits promoting-tumour activity through upregulating GPX4 to diminish ferroptosis in hepatocellular carcinoma
Source: Epigenetics. 2023 Mar 29;18(1):2192438. doi: 10.1080/15592294.2023.2192438 (PMC10064926; doi:10.1080/15592294.2023.2192438)

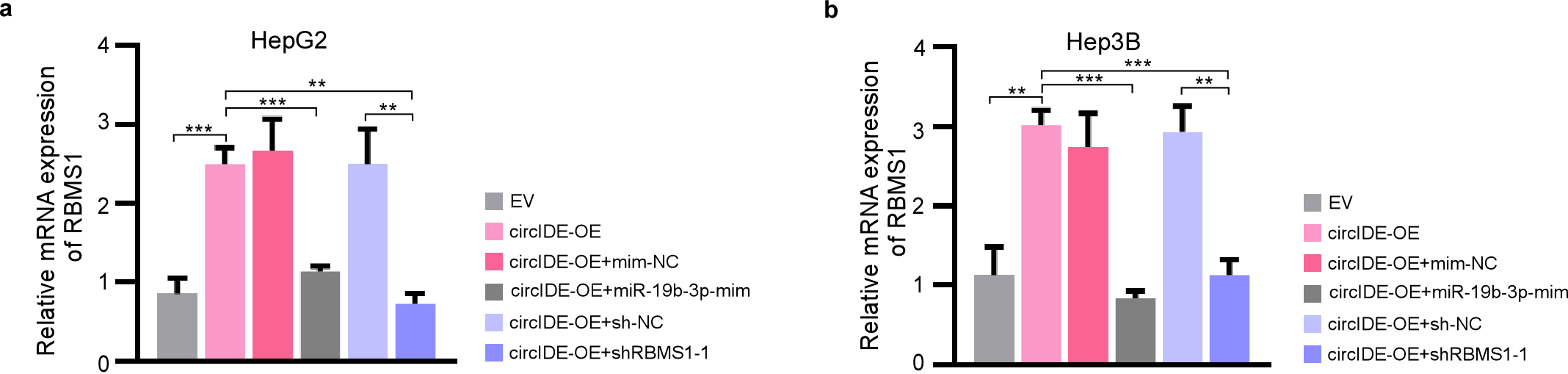

Supplement: Supplemental Material [file KEPI_A_2192438_SM0479.zip › Supplementary files/Supplementary Figure S1 RBMS1.tif]
